# Discovery of an ApoE4‐Targeted Small‐Molecule SirT1 Enhancer for the Treatment of Alzheimer’s Disease

**DOI:** 10.1002/alz70859_101293

**Published:** 2025-12-25

**Authors:** Samar Padder, Jesus J Campagna, Sujyoti Chandra, Bruce Teter, Whitaker Cohn, Johnny Pham, Youngsuk Song, Barbara Jagodzinska, Kanagasabai Vadivel, Mohammad Parvez Alam, Tina Bilousova, Malaney Young, Chris Jean Elias, Juan Marcucci, Ilinca Flacau, Ainsley Jackman, Dongwook Wi, Chunni Zhu, Patricia Spilman, Michael Jung, Dale E Bredesen, Varghese John

**Affiliations:** ^1^ University of California, Los Angeles (UCLA), Los Angeles, CA USA

## Abstract

**Background:**

Alzheimer’s disease (AD) is characterized by amyloid plaques and tau tangles, with apolipoprotein E4 (ApoE4) recognized as a strong genetic risk factor for sporadic AD. ApoE4 has been shown to repress the expression of Sirtuin 1 (SirT1), a key neuroprotective protein, contributing to disease progression. This study describes the discovery and preclinical evaluation of DDL‐218, a small‐molecule SirT1 enhancer targeting ApoE4 to mitigate its repressive effects on SirT1.

**Method:**

A high‐throughput screening in N2a‐apoE4 cell line identified SirT1 enhancer candidate compounds. Lead compounds were identified through medicinal chemistry and in silico modeling. In vitro testing in ApoE4‐expressing neuronal cells and in vivo acute and chronic studies using ApoE4(TR):5xFAD transgenic AD mouse models were conducted. Protein interactions were examined through affinity purification, proteomics, and chromatin immunoprecipitation. Gene expression changes in SirT1, NYFB and PRMT5 were measured by qRT‐PCR. Memory improvement was assessed using Barnes Maze test. Hippocampal tissue from DDL‐218‐ and vehicle‐treated mice underwent global proteomics and thermal proteome profiling (TPP) to identify differentially expressed proteins and elucidate the mechanism of action of DDL‐218.

**Result:**

DDL‐218 significantly increased SirT1 protein and mRNA levels in neuronal cells by upregulating transcription factor NFYB and enzyme PRMT5. Drug treatment led to displacement of ApoE4 from the SirT1 promoter, allowing enhanced SirT1 expression. In AD model mice, DDL‐218 treatment improved memory performance observed in the Barnes Maze test, and enhanced SirT1, NFYB, and PRMT5 mRNA in the brain. Proteomics revealed that DDL‐218 upregulated proteins associated with neuronal function, including PTprn2, which plays a role in synaptic plasticity. Additionally, DDL‐218 showed favorable brain penetration and no observable adverse effects.

**Conclusion:**

DDL‐218 successfully enhanced SirT1 expression by counteracting ApoE4’s repressive effects, demonstrating potential as a therapeutic strategy for AD. DDL‐218 improved memory and key neural pathways, showing promise in preclinical models. The observed cognitive improvements in the mouse model support further investigation of DDL‐218 as a novel treatment for Alzheimer’s, targeting ApoE4‐driven disease mechanisms. Future studies will establish protein targets of DDL‐218 and clarify its mechanism in increasing NFYB mRNA levels. Additionally, we will evaluate its safety profile and efficacy in other ApoE4‐expressing in vivo models.

